# TDP-43 pathology is associated with divergent protein profiles in ALS brain and spinal cord

**DOI:** 10.1186/s40478-025-02084-y

**Published:** 2025-08-18

**Authors:** Emily Feneberg, Alexander G. Thompson, Philip D. Charles, Iolanda Vendrell, Benedikt M. Kessler, Roman Fischer, Olaf Ansorge, Elizabeth Gray, Kevin Talbot, Martin R. Turner

**Affiliations:** 1 Department of Neurology, Technical University of Munich School of Medicine and Health, Munich, Germany; 2 Nuffield Department of Clinical Neurosciences, University of Oxford, Oxford, UK; 3 Target Discovery Institute, Centre for Medicines Discovery, Nuffield Department of Medicine, University of Oxford, Oxford, UK; 4 John Radcliffe Hospital, West Wing Level 6, Oxford, OX3 9DU UK

**Keywords:** TDP-43, Amyotrophic lateral sclerosis, Proteomics, Tissue, Brain, Spinal cord

## Abstract

Neuronal and glial cytoplasmic inclusions positive for TAR DNA-binding protein 43 (TDP-43) are the defining pathological hallmark of 97% of amyotrophic lateral sclerosis (ALS) and 50% of frontotemporal dementia (FTD). The ALS-FTD clinicopathological spectrum variably involves cortical and spinal anterior horn cell pathology. The broader protein composition of these inclusions is of major importance to understanding pathogenesis, clinical heterogeneity and biomarker development. This study examined the proteome associated with TDP-43 inclusions in ALS, using mass spectrometry-based proteomic analysis of spinal cord and cerebral cortex from donors with phosphoTDP-43 positive ALS (*n* = 16), alpha-synuclein positive Parkinson’s disease (PD, *n* = 8), phosphotau and beta-amyloid positive Alzheimer’s disease (AD, *n* = 8) and age matched non-neurological controls (*n* = 8), comparing ALS with non-ALS conditions, spinal cord with cerebral cortex samples, and detergent-soluble with -insoluble fractions. Increased abundance of TDP-43 in the detergent-insoluble fraction of ALS cortex and spinal cord tissue confirmed disease-specific protein enrichment by serial fractionation. The most striking alterations between ALS and other conditions were found in the detergent-insoluble fraction of spinal cord, with predominant enrichment of endosomal and extracellular vesicle pathways. In the cortex mitochondrial membrane/envelope and ion transmembrane transport pathways were enriched in the detergent-insoluble fraction. RNA/DNA metabolic processes (in spinal cord) versus mitochondrial and synaptic protein pathways (in cortex) were upregulated in the detergent-soluble fraction of ALS cases and downregulated in the insoluble protein fraction. Whilst motor cortex and spinal cord may not optimally reflect disease-specific pathways in AD, in PD a significant enrichment of alpha-synuclein in the detergent-insoluble fraction of spinal cord was found. Among proteins concordantly elevated in the detergent-insoluble fractions of spinal cord and cortex, there was greater representation of proteins encoded by ALS-associated genes, specifically Cu/Zn superoxide dismutase 1, valosin containing protein and TDP-43 (odds ratio 16.34, *p* = 0.002). No significant increase in TDP-43 interacting proteins was observed in either detergent-soluble or -insoluble fractions. Together, this study shows a divergence in the composition of proteins associated with TDP-43 positive detergent-insoluble inclusions between spinal cord and cerebral cortex. A common upregulation of proteins encoded by ALS-causing genes implicates their role in the pathogenesis of the ALS-FTD spectrum of diseases beyond TDP-43. Data are available via ProteomeXchange with identifier PXD067060.

**Supplementary Information:**

The online version contains supplementary material available at 10.1186/s40478-025-02084-y.

## Introduction

Detergent-insoluble proteinaceous inclusions are the unifying pathological feature of neurodegenerative diseases, with differences in protein composition and regional distribution related to clinical phenotype. Key constituent proteins in these inclusions have been genetically and mechanistically linked to disease pathogenesis, though the extent to which protein aggregation is causative of neuronal loss or reflects a compensatory mechanism, for example serving to sequester toxic proteins, is debated. Ubiquitinated neuronal and glial cytoplasmic aggregates of the 43KDa transactive response DNA and RNA binding protein, TDP-43, are the histopathological hallmark in 97% of ALS cases [28], with a strong predilection for the motor cortex and its frontal connectome, and the spinal cord anterior horns. In most apparently sporadic ALS cases, TDP-43 pathology consists of large cytoplasmic inclusions and relatively few dystrophic neurites. TDP-43 pathology is also found in 50% of frontotemporal lobar degeneration (FTLD), most commonly associated with the clinical syndrome of behavioural variant frontotemporal dementia (bvFTD), which has clinical overlap with ALS [3]. Rare variants in the TARDBP gene, encoding TDP-43, located within the aggregation-prone c-terminal low complexity region, are a rare cause of ALS, providing a mechanistic link between TDP-43 dysregulation and ALS. Interest in TDP-43 proteinopathies has broadened since the identification of predominant TDP-43 neuropathology in 30% of Alzheimer’s dementia cases, leading to re-classification of these cases as limbic-predominant age-related TDP-43 encephalopathy (LATE) [27].

Aggregated TDP-43 pathology in *post mortem* tissue has been well-defined by neuropathological studies or biochemical analysis of the urea soluble tissue fraction, where typical characteristics are ubiquitination, phosphorylation and truncation of TDP-43 [28]. Mass spectrometry analysis of human ALS or FTD tissue has been limited to unfractionated brain homogenates rather than TDP-43 inclusion-specific fractions and has therefore predominantly revealed alterations linked to pathways reflecting neurodegeneration and inflammation more broadly [29, 38, 40]. In addition there has been no direct comparison of the cortex and spinal cord tissue to understand regional differences in ALS [12, 23, 29].

Determining protein constituents associated with detergent-insoluble inclusions that are specific to TDP-43 proteinopathies compared with other proteinopathies, for example those seen in Parkinson’s disease (PD) or Alzheimer’s disease (AD), could increase understanding of the wider consequences of TDP-43 aggregation on ALS pathogenesis, identify druggable pathways, and aid the development of specific biomarkers to stratify patients by TDP-43 molecular diagnosis.

This study used an established protocol based on increasingly stringent solubilisation to extract proteins in different states of aggregation from *post mortem* tissue samples of people with ALS. Comparing detergent-soluble and -insoluble fractions with other disease conditions and with non-neurological control samples, this study sought to understand the TDP-43 specific aggregated proteome in terms of enriched pathways, the relevance of proteins that interact with TDP-43 in the non-disease state and look for the presence of proteins encoded by genes associated with ALS.

## Methods and materials

### Human samples

Insoluble (urea) and soluble (sarkosyl) protein fractions from primary motor cortex (BA4) and spinal cords from individuals diagnosed with ALS, PD, AD, and controls (CTL) obtained from the Oxford Brain Bank were used [16]. For 3/16 ALS and 3/8 PD cases, motor cortex was not available and dorsolateral prefrontal cortex (BA9/46) was used instead. Patients who donated tissue for this study reported no family history but were not formally genotyped. Donors had provided written informed consent for brain donation and the use of the material and clinical information for research purposes under Research Ethics Committee approval (REC 15/SC/0639, HTA license 12217).

### Sample preparation for mass spectrometry

Fractionation of tissue was done as described previously, with modifications of the protocol described by Neumann and colleagues and stored at -80 °C until use [16, 28]. Samples were thawed on ice and reduced with 5mM dithiothreitol (Alfa Aesar) followed by alkylation with 20mM iodoacetamide (IAA). Samples were subsequently precipitated by methanol-chloroform extraction. Pellets were fully resuspended by addition of 6M urea (Merck) and brief sonication. 50mM triethylammonium bicarbonate (TEAB) (Merck) was added to reduce the final concentration of urea to 1M. The samples were incubated with 2µg trypsin (Promega) at 37 °C, on a shaker for 14–16 h. The reaction was stopped by addition of formic acid (Millipore). Samples were desalted using solid phase extraction (SOLA SPE) columns (ThermoFisher). All in-solution samples were dried in a speed-vac (ThermoScientific) and pellets were resuspended in 2% acetonitrile with 0.1% formic acid diluted in MilliQ water.

### LC-MS/MS data acquisition

Soluble and insoluble protein fraction were analysed by liquid chromatography tandem mass spectrometry (LC-MS/MS) using an Evosep OneLC coupled to a Bruker timsTOF Pro mass spectrometer (OtofControl 6.0.115/HyStar 5.0.37.1) as described previously in DDA-PASEF mode [21]. TimsTOF raw data was searched in Maxquant 1.6.14. against a reviewed Homo sapiens Uniprot database (retrieved April 2019). Maxquant default settings were used with oxidation of methionine residues and acetylation of protein N-termini set as variable modifications and carbamidomethylation of cysteine residues as fixed modification. The match between runs feature was used for all analyses. Deidentified raw data and Maxquant search results were uploaded to the public data repository PRIDE [31] with the identifier number. The mass spectrometry proteomics data have been deposited to the ProteomeXchange Consortium via the PRIDE [30] partner repository with the dataset identifier PXD067060.

### Data analysis

Bioinformatic data analysis was conducted using R (R-studio Version 1.1.383). To further improve confidence in protein identification at least two unique peptides were required for protein identification, with 0.1% peptide false discovery rate (FDR) and 1% protein FDR. Protein abundance values were normalised to the median abundance of the 90% of proteins with the lowest variance across all runs [22]. Proteins with > 50% missing values were excluded. Missing data were not imputed. Abundances were compared by t-test and p-values adjusted for multiple comparisons using the Benjamini-Hochberg FDR. Individual comparisons of the pathology-associated proteins alpha-synuclein, tau and TDP-43 between groups were not corrected for multiple comparisons. Comparisons across tissue types and detergent-soluble and -insoluble fractions were performed for proteins overlapping between contrasts, considering proteins for which the product of fold change in both tissues or fractions was greater than 1.5. Gene ontology (GO) overrepresentation analysis was performed using ClusterProfiler [42], with the foreground of uniquely identified proteins in each fraction or the overlapping proteome compared with a background of all proteins identified in both detergent-soluble and -insoluble fractions; GO enrichment analysis was performed ranking proteins by the product of fold change and -log_10_ p-value. GO encrichment and overrepresentation analyses were adjusted by FDR. Overrepresentation analysis for TDP-43 interactor proteins was performed using TDP-43 interactors identified in Feneberg et al. [14].

## Results

### Comparison of *post mortem* tissue cohort specimens

Protein fractionations from spinal cord, motor cortex (in the region of the ‘hand knob’) or dorsolateral prefrontal cortex were used from our previous study (Table 1 and Supplementary File S1) [16]. The AD group was significantly older than control, ALS and PD groups. *Post mortem* time from death to fixation was similar between groups (*p* = 0.6). ALS patients who donated tissue for this study were apparently sporadic cases, not reporting a relevant family history.

**Table 1:** Characteristics of the *post mortem* tissue cohort

Diagnostic group	Cortex, *n*^a^ (Motor/Frontal)	Spinal Cord, *n* (Thoracic/Cervical/Lumbar)	Mean (range) age (years)	Gender (f/m) ^b^
Control	8 (8/0)	8 (7/1/0)	65.9 (38–89)	(4/4)
PD	8 (5/3)	6 (6/0/0)	76.9 (69–80)	(1/7)
AD	8 (8/0)	8 (8/0/0)	84.3*** (78–93)	(4/4)
ALS	16 (13/3)	16 (12/3/1)	65.6 (49–86)	(6/10)

*** Increased compared to all other groups (*p* < 0.001)

^a^ n = number.

^b^ f = female, m = male.

PD – Parkinson’s disease; AD – Alzheimer’s disease; ALS – amyotrophic lateral sclerosis

### Enrichment of pathological TDP-43

We previously performed serial fractionation of cortex and spinal cord tissue from people with ALS, PD, AD and controls to obtain insoluble protein fractions to investigate the pathological proteome associated with TDP-43 proteinopathy compared to other proteinopathies (AD and PD) [16]. The ALS detergent-insoluble fraction showed the typical TDP-43 characteristics with smaller c-terminal fragments at 25 kDa and 35 kDa by immunoblotting with an anti-C-terminal TDP-43 antibody which were not found in AD or PD.

### TDP-43-specific proteome enrichment clusters by cortex and spinal cord region

LC-MS/MS data from the detergent-insoluble fraction identified 4052 proteins in total (Supplementary Table S1). After filtering for proteins that were present in more than 50% of samples, 1398 proteins were analysed. In the detergent-soluble fraction, 4192 proteins were identified in total. After filtering for proteins that were present in more than 50% of samples, 3257 proteins were analysed. Hierarchical clustering of protein abundances from detergent-soluble and -insoluble fractions showed that samples clustered by region (cortex versus spinal cord) rather than disease groups (ALS, AD, PD and control) (Supplementary Fig. S1).

### TDP-43 accumulates in the detergent-insoluble protein fraction of the brain and spinal cord

**Fig. 1:**
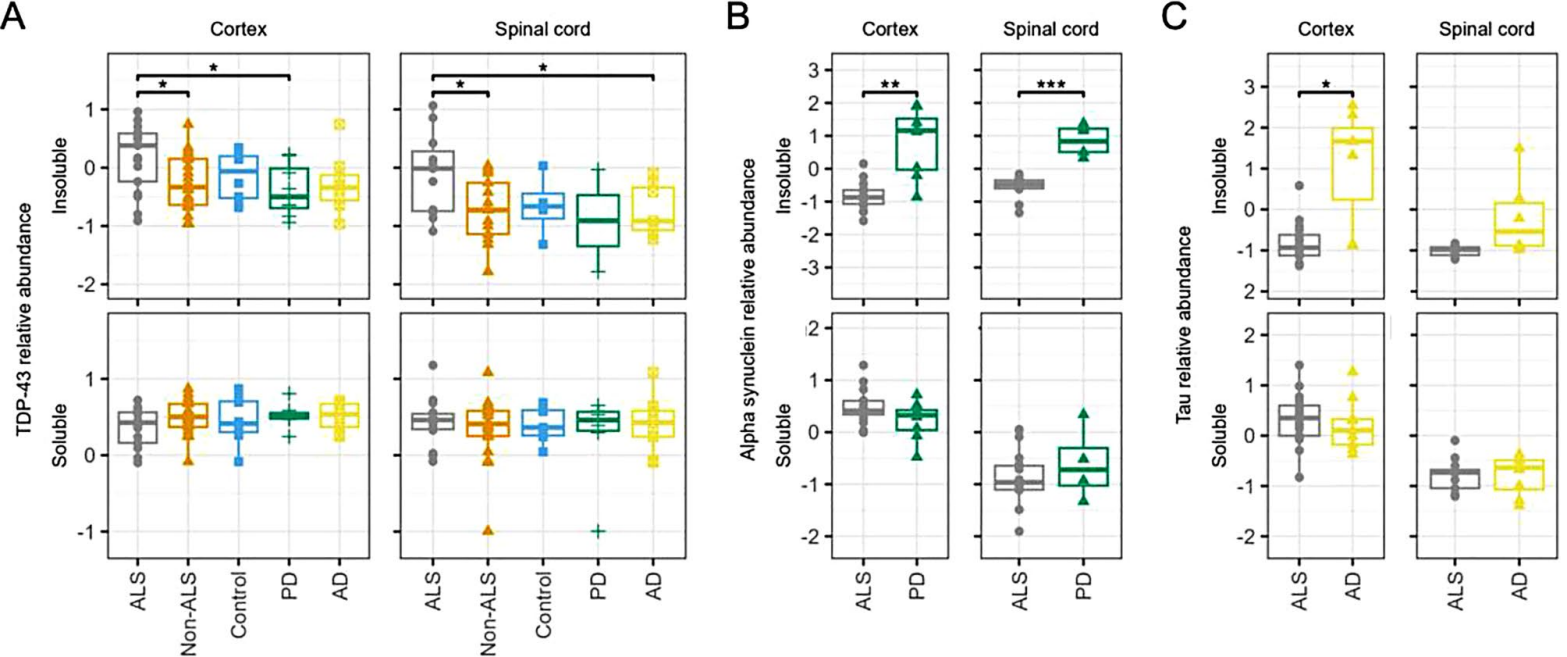
Abundance of disease-associated proteins in detergent-soluble and -insoluble fractions. **A** Abundance of TDP-43 in ALS compared to non-ALS samples in the detergent-insoluble and detergent-soluble fractions shows enrichment of aggregate-specific TDP-43 in ALS cortex and spinal cord within the insoluble but not soluble fractions. **B** - **C** Abundance of disease aggregate-associated proteins alpha-synuclein and tau demonstrates enrichment of alpha-synuclein in detergent-insoluble, but not detergent-soluble fractions from people with PD compared with ALS (**B**) and tau in detergent-insoluble, but not detergent-soluble fractions from people with AD compared with ALS (**C**). PD – Parkinson’s disease; AD – Alzheimer’s disease; ALS – amyotrophic lateral sclerosis.

Characteristic ALS, PD and AD disease-associated proteins were identified in the detergent-insoluble fraction (Supplemenatry Table S1). Comparing relative abundance of these proteins between groups indicated higher levels of TDP-43 in the detergent-insoluble protein fraction of cortex from people with ALS when compared with all other conditions (ALS vs. all log_2_ fold change, fc = 0.45, unadjusted *p* = 0.012). Differences in TDP-43 abundance comparing ALS with individual disease groups indicated a consistent direction of change in ALS, but this was only significant comparing samples from people with ALS with PD (ALS versus non-ALS fc = 0.45, unadjusted *p* = 0.012; ALS versus control fc = 0.32, unadjusted *p* = 0.136; ALS versus PD fc = 0.57, unadjusted *p* = 0.017; ALS versus AD fc = 0.46, unadjusted *p* = 0.062). Similar findings were observed for spinal cord (ALS versus non-ALS fc = 0.63, unadjusted *p* = 0.029; ALS versus control fc = 0.55, unadjusted *p* = 0.160; ALS versus AD fc = 0.62, unadjusted *p* = 0.039; there was insufficient data for ALS versus PD as TDP-43 was only detected in a subset of PD samples; Fig. 1A). Notably, differences in TDP-43 abundance were not detected when correcting for multiple comparisons.

Levels of α-synuclein, a major constituent of PD-associated protein aggregates, had lower abundance in the detergent-insoluble fraction of cortex from people with ALS compared with PD (fc = -1.65, unadjusted *p* = 0.002; Fig. 1B). Tau, the major constituent of AD-associated neurofibrillary tangles, had lower abundance in detergent-insoluble cortex from people with ALS compared with AD (fc = -1.92, unadjusted *p* = 0.011). Similar differences in α-synuclein (ALS versus PD fc = -1.43, unadjusted *p* < 0.001) but not tau levels (ALS versus AD fc = -0.82, unadjusted *p* = 0.094) were observed in the detergent-insoluble fraction from spinal cord (Fig. 1C).

In the detergent-soluble fraction, levels of TDP-43 were similar to other conditions (cortex ALS versus all fc = -0.12, unadjusted *p* = 0.107; spinal cord ALS versus all fc = 0.08, unadjusted *p* = 0.547; Fig. 1A). Abundance of α-synuclein (ALS versus PD cortex fc = 0.25, unadjusted *p* = 0.130; spinal cord fc = -0.28, unadjusted *p* = 0.500) and tau (ALS versus AD cortex fc = 0.12, unadjusted *p* = 0.593; spinal cord fc = 0.01, unadjusted *p* = 0.962) were similar between conditions in the detergent-soluble fraction of both spinal cord and cortex (Fig. 1B - C).

Together, these results indicated successful enrichment of aggregate-associated proteins, specifically in the detergent-insoluble fraction.

In the urea fraction, twelve peptides of the N- and C-terminal end of TDP-43 were detected (Table 2). Five peptides were located after previously reported [16] N-terminal truncation sites at 175-(K)LPNSKQS QDEPL and at 266-SNRQLERSG (R)F. In the soluble fraction, 10 peptides were identified, four after the truncation sites and one covering the first truncation site at AA 175.

**Table 2:** List of TDP-43 peptides identified in the insoluble and soluble protein fraction

Sequences within TDP-43	AA Start	AA End	Insoluble	Soluble
VTEDENDEPIEIPSEDDGTVLLSTVTAQFPGACGLR	7	42	x	x
LVEGILHAPDAGWGNLVYVVNYPK	56	79	x	x
LVEGILHAPDAGWGNLVYVVNYPKDNKR	56	83	x	
KMDETDASSAVK	84	95	x	
TSDLIVLGLPWK	103	114	x	x
TTEQDLKEYFSTFGEVLMVQVK	115	136	x	x
FTEYETQVK	152	160	x	x
WCDCKLPNSK	172	181		x
CTEDMTEDELREFFSQYGDVMDVFIPKPFR	198	227	x	x
EFFSQYGDVMDVFIPKPFR	209	227	x	
AFAFVTFADDQIAQSLCGEDLIIK	228	251	x	x
GISVHISNAEPK	252	263	x	x
FGGNPGGFGNQGGFGNSR	276	293	x	x

AA - amino acid

*Comparison of ALS*,* AD and PD with other conditions*

Comparison of the proteome of different fractions of spinal cord and cortex from people with ALS, AD or PD against all other conditions was performed. Differential abundance for all proteins analysed in all contrasts (including ALS, AD and PD vs. individual disease and control groups) and full GO enrichment results are provided in Supplementary Tables S1 and S2.

**Fig. 2:**
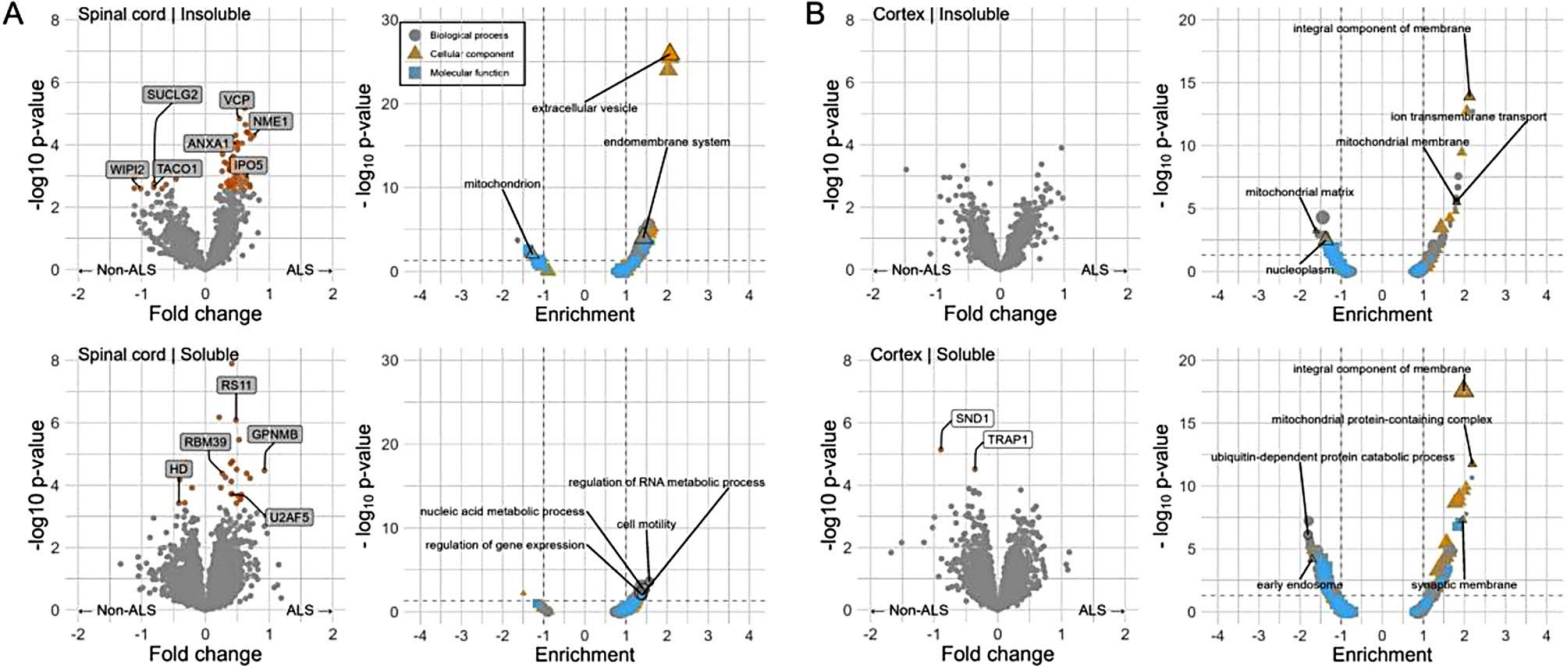
Differential abundance of proteins and gene ontology enrichment analysis. In samples from spinal cord (**A**) and cortex (**B**) insoluble and soluble fractions from people with ALS compared with other groups. Yellow points indicate FDR-adjusted p ≤ 0.05. ALS – amyotrophic lateral sclerosis.

The most marked changes were observed comparing the detergent-insoluble fraction of ALS spinal cord with other conditions. A large number of differentially abundant proteins (FDR-adjusted *p* ≤ 0.05) were identified: 56 increased and 8 decreased (Fig. 2A). Upregulated proteins included importin IPO5 (fc = 0.6, FDR-adjusted *p* = 0.044), NMDA receptor component NME1 (fc = 0.8, FDR-adjusted *p* = 0.007), valosin containing protein (VCP), rare variants of which are associated with ALS, FTD and multisystem proteinopathy [4, 20] (fc = 0.5, FDR-adjusted *p* = 0.007) and inflammatory protein ANXA1 (fc = 0.5, FDR-adjusted *p* = 0.008). Downregulated were the neurofilament light protein NEFL (fc = -0.46, FDR-adjusted *p* = 0.001), mitochondrial proteins including SUCLG2 (fc = -0.8, FDR-adjusted *p* = 0.041) and TACO1 (fc = -0.8, FDR-adjusted *p* = 0.043) and the autophagy protein WIPI2 (fc = -1, FDR-adjusted *p* = 0.046).

Gene ontology enrichment analysis indicated marked enrichment of terms (FDR-adjusted *p* ≤ 0.05) relating to extracellular vesicle, extracellular exosome and the endosome, regulation of the stress response and apoptosis. Negative enrichment was observed for terms related to the mitochondrion and adenyl ribonucleotide binding.

**Fig. 3:**
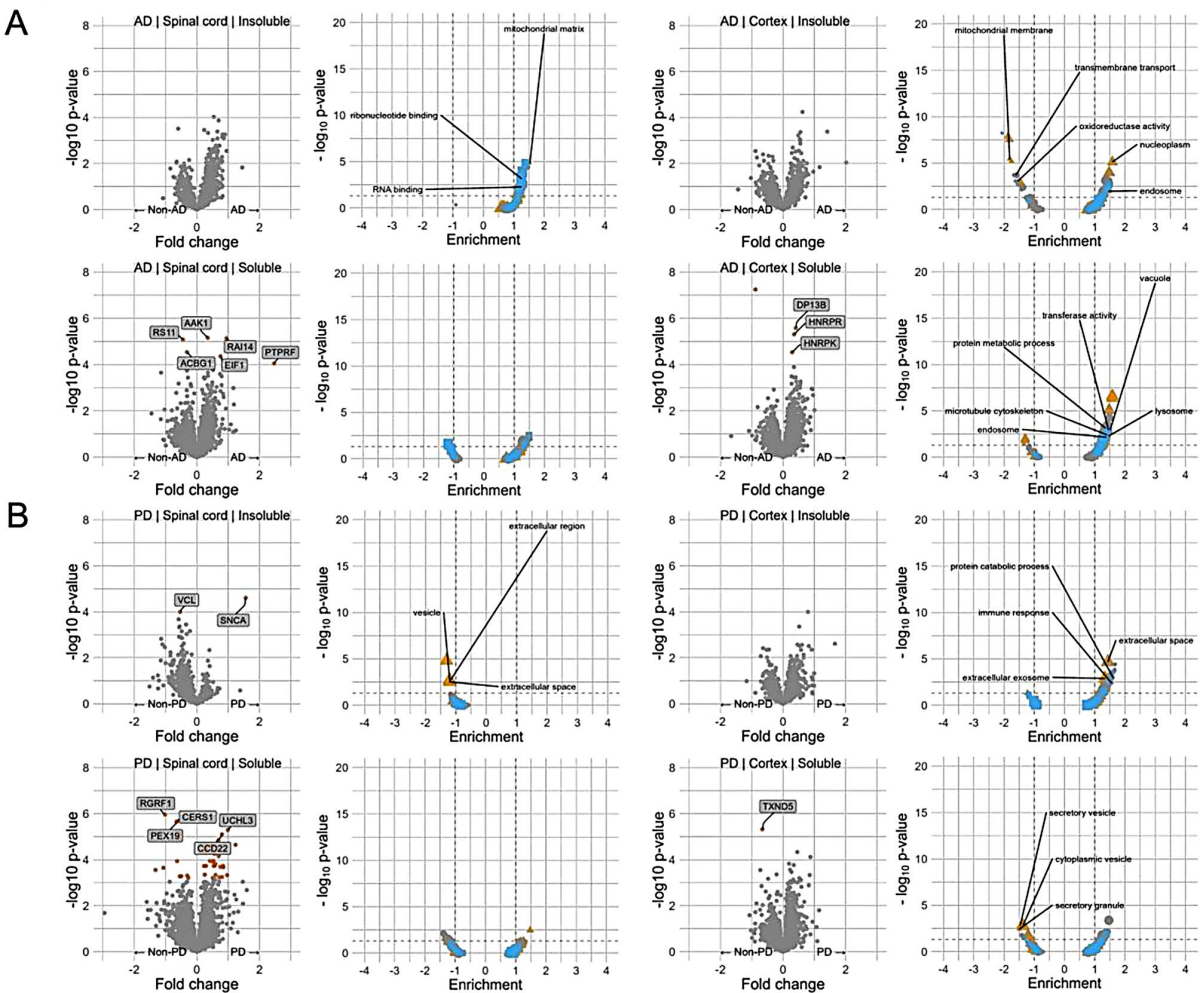
Differential abundance of proteins and gene ontology enrichment analysis. In samples from spinal cord and cortex insoluble and soluble fractions from people with AD (**A**) and PD (**B**) compared with other groups. Yellow points indicate FDR-adjusted *p *≤ 0.05. AD – Alzheimer’s disease, PD – Parkinson’s disease

In the detergent-insoluble fraction of AD spinal cord no differentially abundant individual proteins were detected after FDR correction. GO enrichment analysis (FDR-adjusted *p* ≤ 0.05) indicated enrichment of terms relating to the mitochondrial matrix and RNA binding (Fig. 3A).

In PD alpha-synuclein was increased in the detergent-insoluble fraction of spinal cord (fc = 1.5, FDR-adjusted *p* < 0.001) and vinculin was decreased (fc = -0.5, FDR-adjusted *p* < 0.001). GO enrichment analysis (FDR-adjusted *p* ≤ 0.05) indicated negative enrichment of extracellular region and vesicle terms (Fig. 3B).

In the detergent-soluble fraction of ALS spinal cord, 26 proteins demonstrated altered abundance: 21 increased, 5 decreased (Fig. 2A). Increased were the ALS biomarker transmembrane glycoprotein GPNMB (fc = 0.9; FDR-adjusted *p* = 0.011), RNA binding protein RBM39 (fc = 0.3; FDR-adjusted *p* = 0.011) and ribosomal protein RS11 (fc = 0.5; FDR-adjusted *p* = 0.001), splicing factor U2AF5 (fc = 0.4; FDR-adjusted *p* = 0.029). Downregulated was Huntingtin (fc = -0.4, FDR-adjusted *p* = 0.0003). GO enrichment analysis (FDR-adjusted *p* ≤ 0.05) indicated enrichment of terms relating to gene expression and regulation of RNA metabolic processes.

In the detergent-soluble fraction of AD spinal cord 7 proteins demonstrated altered abundance. Decreased were the ribosomal protein RS11 (fc = -0.4, FDR-adjusted *p* < 0.001) and the long-chain-fatty-acid-CoA-ligase ACBG1 (fc = -0.3, FDR-adjusted *p* < 0.001). Increased were Ankycorbin RAI14 (fc = 0.9, FDR-adjusted *p* < 0.001), a protein kinase AAK1 (fc = 0.4, FDR-adjusted *p* < 0.001), and elongation factors EIF1 (fc = 0.8, FDR-adjusted *p* < 0.001) and EF1D (fc = 0.5, FDR-adjusted *p* < 0.001) and phosphatase PTPRF (fc = 2.5, FDR-adjusted *p* < 0.001) (Fig. 3A). In the detergent-soluble fraction of PD spinal cord 54 proteins demonstrated altered abundance: 39 increased, 15 decreased. The top proteins were decreased including a ceramide synthase CERS1 (fc = -0.6, FDR-adjusted *p* < 0.001), a ras-specific guanine nucleotide-releasing factor RGRF1 (fc = -1.0, FDR-adjusted *p* < 0.001) and the peroxisomal factor PEX19 (fc = -0.7, FDR-adjusted *p* < 0.001). Increased were the ubiquitin hydrolase UCHL3 (fc = 0.7, FDR-adjusted *p* < 0.001) and the coiled-coil domain protein CCD22 (fc = 0.9, FDR-adjusted *p* < 0.001) (Fig. 3B). There was no enrichment of GO terms (FDR-adjusted, *p* ≤ 0.05) in AD or PD fractions.

In the detergent-insoluble fraction of ALS cortex no differentially abundant individual proteins were detected after FDR correction (Fig. 2B), though there was enrichment of GO terms (FDR-adjusted *p* ≤ 0.05) relating to the mitochondrial membrane and envelope, transmembrane, ion transmembrane transport and negative enrichment of terms related to nucleoplasm and mitochondrial matrix.

In the detergent-insoluble fraction of AD cortex no differentially abundant individual proteins were detected after FDR correction. Negative enrichment of GO terms related to mitochondrial membrane and envelope and transmembrane transport. Positive enriched were the terms nucleoplasm and endosome (Fig. 3A). In PD no differentially abundant individual proteins were detected after FDR correction. Enrichment of GO terms related to extracellular space, exosome and immune response (Fig. 3B).

In the detergent-soluble fraction of ALS cortex, there were 2 proteins downregulated, the endonuclease SND1 that mediates miRNA decay and is involved in STAT6 pathway (fc = -0.9, FDR-adjusted *p* = 0.022) and the mitochondrial protein TRAP1 (fc = -0.4, FDR-adjusted *p* = 0.047) (FDR-adjusted *p* ≤ 0.05) (Fig. 2B). GO enrichment analysis (FDR-adjusted *p* ≤ 0.05) indicated enrichment of terms with relating to the mitochondrion and synapse, with negative enrichment of terms related to the proteasome, endosome and protein ubiquitination.

In the detergent-soluble fraction of AD cortex 4 proteins demonstrated altered abundance: 3 increased, 1 decreased: Two heterogeneous nuclear ribonucleoproteins, HNRPR (fc = 0.4, FDR-adjusted *p* < 0.001) and HNRPK (fc = 0.3, FDR-adjusted *p* < 0.001), and the DCC-interacting protein DP13B (fc = 0.4, FDR-adjusted *p* < 0.001) were increased and activating signal activator ASCC3 (fc = 0.9, FDR-adjusted *p* < 0.001) was decreased. GO enrichment analysis (FDR-adjusted *p* ≤ 0.05) indicated enrichment of terms relating to the vacuole and transferase activity, the lysosome and endosome and protein metabolic processes (Fig. 3A). In PD TXND5 was decreased (fc = -0.7, FDR-adjusted *p* < 0.001) and GO terms negatively enriched related to vesicles (Fig. 3B).

### Comparison of soluble and insoluble fractions between cortex and spinal cord

**Fig. 4:**
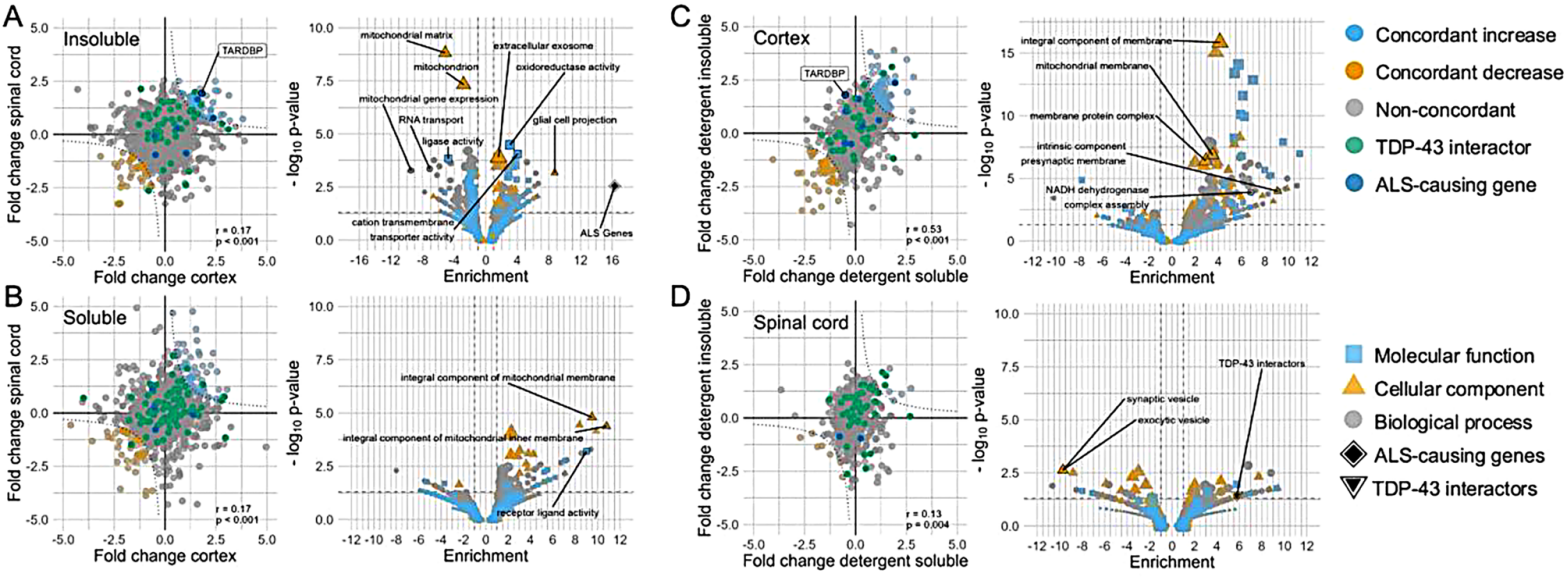
Comparison of alterations in ALS spinal cord and cortex in detergent-soluble and detergent-insoluble fractions. **A-B** Comparison of fold change and gene ontology enrichment analysis in concordantly up- and downregulated proteins in detergent-insoluble (**A**) and -soluble (**B**) fractions across tissue types. **C-D** Comparison of fold change and gene ontology enrichment analysis in concordantly up- and downregulated proteins in cortex (**C**) and spinal cord (**D**) samples across different fractions. Dashed lines indicate areas of concordant differential abundance. Green points indicate TDP-43 interacting proteins, dark blue points indicate proteins encoded by ALS-causing genetic variants. Black rhombus and triangle indicate overrepresentation analysis of proteins encoded by ALS-causing genetic variants and TDP-43 interactors within those proteins concordantly upregulated. ALS – amyotrophic lateral sclerosis; TARDBP – TDP-43

Due to the difference in matrix limiting direct comparisons of abundance between detergent-soluble and -insoluble fractions, and of spinal cord and cortex, it was not possible to directly compare quantitative mass spectrometry data. Instead, comparison within soluble and insoluble fractions across tissues was undertaken by comparing fold change for overlapping proteins between ALS cases and all other groups to identify shared alterations in the same fraction from different regions. This indicated weak correlation of abundance differences in cortex and spinal cord (detergent-insoluble *r* = 0.17, unadjusted *p* < 0.001; detergent-soluble *r* = 0.17, unadjusted *p* < 0.001; Fig. 4A-B, Supplementary Table S3).

In the detergent-insoluble fraction, 51 proteins demonstrated concordant upregulation and 38 concordant downregulation in the cortex and spinal cord (Fig. 4A). Proteins of interest that were upregulated in the insoluble fraction were Cullin-RING E3 ubiquitin protein CAND1, the Rab protein GDP-dissociation inhibitor GDI2, oxidative stress proteins, Glutathione-S transferase LANC1 and PRDX6, amine oxidase AOFB, mitochondrial MDH2, pyruvate kinase and transcription regulator (e.g. STAT3) PKM, Ras-related protein RAP1A, histone deacetylase SIRT2 and the ALS protein VCP of the ternary complex to export ubiquitinated proteins from the ER to cytoplasm. Significantly downregulated were the nuclear export protein XPO1, mitochondrial proteins SUCLG2 and MMUT and the acetyl-coenzyme A synthetase ACSS1. GO overrepresentation analysis indicated enrichment of extracellular exosome and transmembrane transporter proteins in the concordantly upregulated proteins in the insoluble fraction. There was overrepresentation of terms related to the mitochondrion, RNA transport and localization, and cellular response to oxidative stress in concordantly downregulated proteins.

There was striking overrepresentation of proteins encoded by ALS-associated genes in the proteins with concordant upregulation in insoluble fractions (Cu/Zn superoxide dismutase, TDP-43 and valosin containing protein, OR 16.34, unadjusted *p* = 0.002; Fig. 4A). However, proteins concordantly upregulated in the insoluble fraction were not enriched for TDP-43 interacting proteins (OR 2.11, unadjusted *p* = 0.147; Fig. 4A).

In the detergent-soluble fraction, 56 proteins demonstrated concordant upregulation and 44 concordant downregulation in the cortex and spinal cord (Fig. 4B). GO overrepresentation analysis of concordantly upregulated proteins showed enrichment of terms related to mitochondria, specifically the inner mitochondrial membrane and included proteins such as peroxidase PRDX3, phosphatase PGAM5, mitochondrial fission protein FIS1 and Lamin-B1 (LMNB1). Proteins concordantly downregulated included transportin 1 (TNPO1), exportin 7 (XPO7), and ubiquitin associated proteins (UBA1, UBE2O, UBR1), though there was no significant GO term overrepresentation in concordantly downregulated proteins. Concordant upregulated proteins in the soluble fractions were not enriched in TDP-43 interactors (OR 1.07, unadjusted *p* = 0.527) or proteins encoded by ALS genes (no overlap; Fig. 4B).

Similar analysis comparing fold changes within tissues but across detergent-soluble and -insoluble fractions was performed. This indicated moderate correlation of differential abundance of proteins in the both fractions in cortex (*r* = 0.53, unadjusted *p* < 0.001) but weak correlation in spinal cord (*r* = 0.13, unadjusted *p* = 0.004; Fig. 4C-D).

In the cortex, 56 proteins were concordantly upregulated, 47 proteins downregulated. Mitochondrial, transmembrane transporter, and synaptic proteins were overrepresented in the proteins with concordant higher abundance in cortex (Fig. 4C). Among upregulated proteins in the cortex were Aquaporin 4 (AQP4), glutamate receptor GRIA2, ubiquinones (e.g. NDUFA11), synaptotagmin SYT1 and syntaxin STXA1, and the superoxide dismutase SOD1. Proteins of interest that were downregulated in CTX were ubiquitin recognition factor in ER-associated degradation protein NPLOC4, ubiquitin conjugator UBA1, deubiquitinases UCHL1 (an ALS biomarker) and USP14. Overrepresentation gene ontology analysis (FDR-adjusted *p* ≤ 0.05) showed no significant downregulation of pathways.

In the spinal cord, only eight proteins were concordantly upregulated including annexin ANXA1, the zinc- and- and calcium-binding protein S100B, and the oxidative stress protein PRDX3 (Fig. 4D). Synaptic proteins SYN2 and SYT1, and CAM kinase phosphatase PPM1E were concordantly downregulated. Gene ontology analysis showed downregulation of synaptic and exocytic vesicle pathways, but no significantly upregulated pathways.

There was a small degree of overrepresentation of TDP-43 interactors in the concordant upregulated proteins in spinal cord (OR 5.77, unadjusted *p* = 0.035; Fig. 4D). There was no enrichment of ALS-associated genes in the concordant upregulated proteins in either spinal cord or cortex.

## Discussion

This study employed LC-MS/MS analysis of *post mortem* tissue, uniquely comparing cortex and spinal cord tissue fractions from patients with ALS, other neurodegenerative disorders and controls. The main aim of this study was to explore alterations within the CNS proteome in regions of consistent pathological change in ALS. The majority of tissue was therefore from ALS motor cortex where TDP-43 pathology is most abundant. Other proteomic studies have focused on the prefrontal cortex of ALS [5]. At the same time our study looked at differential changes in AD and PD. The inclusion of different neuroanatomical regions and detergent-soluble and detergent-insoluble fractions permitted a comparison of ALS and non-ALS samples as well as an exploration of consistencies and differences across location and biochemical fraction. This demonstrated expected differences between known disease associated-proteins in detergent-insoluble and -soluble fractions between diseases and highlighted major alterations in protein composition at the individual protein and pathway level in those fractions across regions.

Alterations of known disease-associated proteins were observed in the detergent-insoluble fraction, with higher levels of TDP-43 in spinal cord and cortex of ALS cases compared with other diseases. Higher levels of alpha-synuclein in PD and tau in AD were found confirming the validity of the approach to enrich for disease-specific proteins. Similar changes were not observed in the detergent-soluble fraction. Though TDP-43 was concordantly elevated in detergent-insoluble fractions of both cortex and spinal cord of people with ALS compared with other conditions, it was not the most differentially abundant protein in either tissue region. In contrast, elevated alpha-synuclein in the spinal cord in PD represented the most differentially abundant protein, consistent with previous neuropathological work identifying Lewy body pathology in the spinal cord of people with PD [11, 33].

In ALS concordant elevation of other proteins in the detergent-insoluble fraction of spinal cord and cortex, including those encoded by the ALS genes *SOD1* and *VCP* was also observed. Although it is possible that this reflects co-localisation of these proteins with TDP-43 in insoluble inclusions, due to the lack of spatial resolution with fractionated bulk tissue samples, this cannot be discerned in the current study. Co-incident (though not colocalised) aggregation of SOD1 and TDP-43 has been described in spinal cord motor neurons [39]; other aggregates co-existent with TDP-43 in neurons in ALS are also described, such as dipeptide repeat protein aggregates in *C9orf72* ALS [2]. Alternative explanations for this concordant elevation include that a cellular environment permissive to TDP-43 aggregation might also preferentially lead to aggregation of other proteins such as SOD1 and, in the case of VCP, its presence may reflect its physiological functions in degradation of aggregated proteins, particularly TDP-43 [7, 44].

Beyond individual protein changes, we described concordant and divergent changes across neuroanatomical regions and fractions at the protein and pathway level. Different pathological processing of TDP-43 has been previously described for brain and spinal cord [18]. Across the contrasts, alterations in mitochondrial and synaptic proteins, as well as those involved in gene expression, protein homeostatic mechanisms and extracellular vesicle proteins, were emergent themes.

Proteins relating to mitochondrial function were concordantly upregulated in the soluble proteome, with most marked upregulation in the cortex, but concordantly downregulated in the insoluble proteome. TDP-43 pathology has been associated with mitochondrial dysfunction, which is in turn proposed as a major pathogenic mechanism of ALS [8]. Cytoplasmic mislocalization of TDP-43 triggers its localization to mitochondria and release of mitochondrial DNA [25, 43]. This process leads to mitochondrial destabilization, production of reactive oxygen species, reduced levels of oxidative phosphorylation and mitochondrial membrane permeabilization, and ultimately activation of an inflammatory response through cGAS/STING. TDP-43 specifically interacts with mitochondrial proteins and alters mitochondrial dynamics, and pathological forms of TDP-43 such as TDP-43 fragments have been found in mitochondrial protein fractions [10]. Recent evidence also suggests alterations in axonal mitochondrial transport and respiration in genetic models of ALS [9, 26]. Other proteomic studies have reported altered mitochondrial pathways in ALS [5]. In frontal cortices, mitochondrial proteins in FTLD with TDP-43 proteinopathy were decreased [40]. This has been attributed to neuronal loss in affected brain regions of FTLD, where cortical atrophy is pronounced compared to the motor cortex of ALS.

Proteins related to extracellular vesicle biogenesis and endosomal functioning were upregulated in detergent-insoluble fractions. TDP-43 turnover, aggregation, and toxicity is suggested to depend on endocytosis and not autophagy [24]. In our data we observe an enrichment of RAB5 and RAB7 proteins but not RAB11, which has been shown downregulated upon TDP-43 knock-down [34]. Alteration of endosome axonal transport has been described in ALS and subsequently contributes to an impaired autophagy [36, 41]. Extracellular vesicles carry cell-derived cargo for intercellular communication and may act as an alternative mechanism for the clearance of unwanted proteins [37]. They are proposed as an important pathway for TDP-43 disposal and pathological propagation and TDP-43 detection [6, 13, 15, 19]. Upregulation of extracellular vesicle terms within the insoluble fraction may be indicative of their use by cells to try to excrete aggregated proteins when other degradative methods are overwhelmed.

In addition we found divergent pathways that were enriched in AD and PD. Although motor/prefrontal cortex or spinal cord do not optimally reflect disease-specific pathways in AD, mitochondrial matrix and ribonucleotide binding were upregulated in the insoluble fraction of spinal cord and mitochondrial membrane and transmembrane transport were downregulated in the insoluble fraction of cortex, both in contrast to ALS. In PD strong enrichment of alpha-synuclein in the detergent-insoluble fractions of spinal cord was associated with downregulation of pathways related to vesicles and extracellular matrix, consistent with findings that alpha-synuclein alters vesicle biogenesis and has the potential to disrupt membranes [1, 17, 32, 35].

Limitations of the study are the relatively small number of cases that were examined and the unavailability of a pure motor cortex cohort in ALS. In addition, separation into soluble and insoluble fractions can only be undertaken on bulk tissue, preventing spatial analysis at the cellular or subcellular level. As a proteomic study of *post mortem* alterations it is necessarily descriptive and explorative, with analysis of model systems necessary to derive mechanistic understanding of its findings.

## Conclusion

This study revealed tissue protein alterations in CNS regions affected by TDP-43 pathology in ALS, highlighting major proteomic differences in the soluble and insoluble fractions of these regions in the disease. Though emphasising the core relevance of TDP-43 pathology, the study highlights that accumulation of insoluble protein in ALS is not restricted to TDP-43 and provides evidence for a role for proteins encoded by other ALS genes in this process and other protein pathways. Further work utilising spatially-resolving techniques and techniques that can manipulate protein aggregation using model systems are required to delineate the mechanisms of these findings.

## Supplementary Information

Below is the link to the electronic supplementary material.

Supplementary Material 1

Supplementary Material 2

Supplementary Material 3

Supplementary Material 4

Supplementary Material 5

## Data Availability

The datasets used and/or analysed during the current study available from the corresponding author on reasonable request. The proteomic datasets generated and analysed during the current study are available in the PRIDE repository with the identifier number PXD067060.

## Abbreviations

AA: Amino acid
AD: Alzheimer’s disease
ALS: Amyotrophic lateral sclerosis
bvFTD: Behavioural variant frontotemporal dementia
CNS: Central nervous system
CTL: Control
fc: Fold change
FDR: False discovery rate
FTD: Frontotemporal dementia
FTLD: Frontotemporal lobar degeneration
GO: Gene ontology
LC-MS/MS: Liquid Chromatography with tandem mass spectrometry
PD: Parkinson’s disease
pTau: Phosphorylated protein Tau
pTDP-43: Phosphorylated TAR-DNA binding protein
TDP-43: TAR-DNA binding protein
