# Supplementary material for: TDP-43 pathology is associated with divergent protein profiles in ALS brain and spinal cord
Source: Acta Neuropathol Commun. 2025 Aug 18;13:175. doi: 10.1186/s40478-025-02084-y (PMC12359902; doi:10.1186/s40478-025-02084-y)
Supplement: Supplementary file 1 — Supplementary Material 1 [file 40478_2025_2084_MOESM1_ESM.docx]

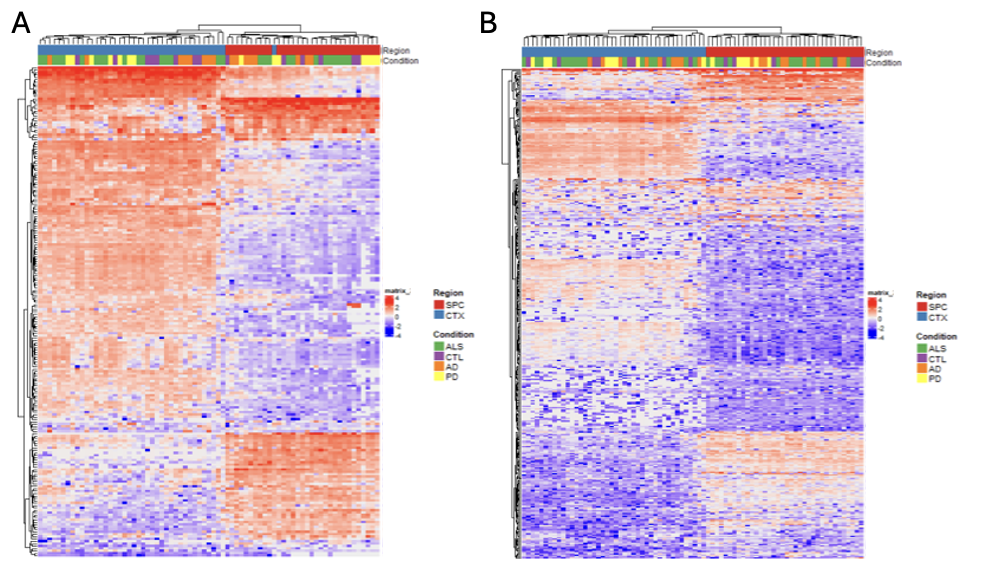


**Supplementary Fig. 1** Hierarchical clustering of protein abundances from detergent soluble and insoluble fractions showed that samples clustered by region (cortex versus spinal cord) rather than disease groups. ALS - amyotrophic lateral sclerosis, AD -Alzheimer’s disease, PD - Parkinson’s disease and CTL – controls. SPC – spinal cord, CTX - cortex
