# Supplementary material for: TDP-43 pathology is associated with divergent protein profiles in ALS brain and spinal cord
Source: Acta Neuropathol Commun. 2025 Aug 18;13:175. doi: 10.1186/s40478-025-02084-y (PMC12359902; doi:10.1186/s40478-025-02084-y)
Supplement: Supplementary file 2 — Supplementary Material 2 [file 40478_2025_2084_MOESM2_ESM.docx]

**Table 1** Detailed clinical and pathology information of the ALS cohort

| Diagnostic group^a^ | Gender  (f/m) | Age  (years) | *Post mortem*  delay (hours) | Disease  duration  (days) | Progression Rate (point/month) | ALSFRS-R | Onset site | El Escorial  criteria | Region  CTX | Region  SPC |
| --- | --- | --- | --- | --- | --- | --- | --- | --- | --- | --- |
| ALS | m | 57 | 48 | 1085 | 0.45 | 29 | RLL | possible | Motor | Thoracic |
| ALS | f | 64 | 48 | 591 | 1.6 | 21 | Bulbar | possible | Motor | Cervical |
| ALS | m | 64 | 24 | 629 | 1.76 | 18 | LUL | probable | Motor | Thoracic |
| ALS | f | 59 | 11 | 849 | 0.82 | 37 | Bulbar | definite | Motor | Thoracic |
| ALS | m | 76 | 96 | 1840 | 0.72 | 19 | Bulbar | definite | Frontal | Cervical |
| ALS | m | 64 | 48 | 1312 | 0.68 | 20 | LUL | definite | Motor | Thoracic |
| ALS | m | 62 | n/a | 1478 | 0.55 | 21 | LLL | probable | Frontal | Cervical |
| ALS | m | 52 | 72 | 1861 | 0.5 | 17 | LLL | definite | Frontal | Cervical |
| ALS | m | 76 | 46 | 389 | n/a | n/a | n/a | n/a | Motor | Thoracic |
| ALS | f | 71 | n/a | 183 | n/a | n/a | n/a | n/a | Motor | Cervical |
| ALS | f | 49 | 57 | 438 | n/a | n/a | Bulbar | n/a | Motor | Thoracic |
| ALS | f | 59 | 11 | 849 | n/a | 37 | Bulbar | n/a | Motor | Thoracic |
| ALS | m | 59 | 24 | 1058.5 | n/a | n/a | n/a | n/a | Motor | Thoracic |
| ALS | m | 86 | n/a | n/a | n/a | n/a | n/a | n/a | Motor | Thoracic |
| ALS | f | 69 | 42 | n/a | n/a | n/a | n/a | n/a | Motor | Thoracic |
| ALS | m | 82 | 25 | 3285 | n/a | n/a | n/a | n/a | Motor | Thoracic |

**^a^** clinical diagnosis according to specialist neurologist. ALS – Amyotrophic lateral sclerosis; m – male; f – female; CTX – cortex; SPC – spinal cord; RLL – right lower limb; LLL – left lower limb; LUL – left upper limb.

**Table 2** Detailed clinical and pathology information of the PD cohort

| Diagnostic group**^a^** | Gender  (f/m) | Age  (years) | *Post mortem*  delay (hours) | Disease  duration  (days) | AD neuropathologic changes**^b^**  “ABC score” | Region  CTX | Region  SPC |
| --- | --- | --- | --- | --- | --- | --- | --- |
| PD | m | 78 | 32 | 4380 | A3, B2, C2. | Motor | Thoracic |
| PD | m | 80 | 24 | 8395 | A1, B1, C0 | Motor | Thoracic |
| PD | m | 79 | 96 | n/a | A0, B1, C0 | Frontal | Thoracic |
| PD | f | 80 | 72 | n/a | A3, B2, C1 | Frontal | Thoracic |
| PD | m | 79 | 84 | 2190 | A1, B1, C0 | Motor | Thoracic |
| PD | m | 69 | 72 | n/a | A3, B1, C0 | Motor | Thoracic |
| PD | f | 80 | 63 | 3285 | A3, B2, C0 | Motor | Thoracic |
| PD | f | 70 | 24 | 6205 | A0, B1, CO | Frontal | thoracic  and  lumbar |

**^a^** clinical diagnosis according to specialist neurologist, **^b^** according to the National Institute on Aging–Alzheimer’s Association guidelines for the neuropathologic assessment of Alzheimer’s disease. PD – Parkinson’s Disease; m – male; f – female; CTX – cortex; SPC – spinal cord.

**Table 3** Detailed clinical and pathology information of the AD cohort

| Diagnostic group**^a^** | Clinical | Gender  (f/m) | Age  (years) | *Post mortem*  delay (hours) | Disease  duration  (days) | AD neuropathologic change**^b^**  “ABC score” | Region  CTX | Region  SPC |
| --- | --- | --- | --- | --- | --- | --- | --- | --- |
| AD | dementia | f | 87 | 68 | 3650 | A3, B3, C3 | Motor | Thoracic |
| AD | mild cognitive impairment | f | 93 | 51 | n/a | A3, B3, C2 | Motor | Thoracic |
| AD | dementia | f | 85 | 29 | 2555 | A3, B3, C3 | Motor | Thoracic |
| AD | dementia | m | 83 | 120 | 1460 | A3, B3, C3 | Motor | Thoracic |
| AD | dementia | m | 85 | 24 | 2190 | A3, B3, C3 | Motor | Thoracic |
| AD | dementia | m | 78 | 34 | 3285 | A3, B3, C3 | Motor | Thoracic |
| AD | dementia | f | 81 | 24 | n/a | A3, B3, C3 | Motor | Thoracic |
| AD | dementia | m | 82 | 65 | 9490 | A2, B2, C1 | Motor | Thoracic |

**^a^** clinical diagnosis according to specialist neurologist, **^b^** according to the National Institute on Aging–Alzheimer’s Association guidelines for the neuropathologic assessment of Alzheimer’s disease. AD – Alzheimer’s Disease; m – male; f – female; CTX – cortex; SPC – spinal cord.

**Table 4** Detailed clinical and pathology information of the CTL cohort

| Diagnostic group^a^ | Gender  (f/m) | Age  (years) | *Post mortem*  delay (hours) | AD neuropathologic change^b^  “ABC score” | Region  CTX | Region  SPC |
| --- | --- | --- | --- | --- | --- | --- |
| CTL | m | 69 | 24 | A0, B0, C0 | Motor | Thoracic |
| CTL | m | 66 | 48 | A0, B1, C0 | Motor | Thoracic /  C junction |
| CTL | f | 89 | 40.5 | A3, B1, C1 | Motor | Thoracic |
| CTL | m | 63 | 83.5 |  | Motor | Thoracic |
| CTL | f | 77 | 70 | A3, B2, C1 | Motor | Thoracic |
| CTL | m | 63 | 24 | A0, B0, C0 | Motor | Cervical |
| CTL | f | 62 | 72 | A0, B0, C0 | Motor | Thoracic |

**^a^** Controls were healthy for age according to clinical diagnosis, **^b^** according to the National Institute on Aging–Alzheimer’s Association guidelines for the neuropathologic assessment of Alzheimer’s disease. CTL – controls; m – male; f – female; CTX – cortex; SPC – spinal cord.
